# Diastereoselective Synthesis of 5-Hydroxy-8-methoxy-1-oxaspiro[5,5]undeca-7,10-diene-9-one

**DOI:** 10.3390/molecules18011174

**Published:** 2013-01-17

**Authors:** Guy L. Plourde, Thomas W. Scully

**Affiliations:** Department of Chemistry, University of Northern British Columbia, 3333 University Way, Prince George, BC V2N 4Z9, Canada

**Keywords:** synthesis, diastereoselective, spirolactone, spiroether, spirocompound, dienone, oxidation, lead tetraacetate

## Abstract

A short five steps synthesis of the title compound from vanillin is described. The racemic spiroether **7** was obtained in 61% yield and in >99% diastereomeric excess (by ^1^H-NMR) from the corresponding phenolic derivative **3** by oxidation with lead (IV) acetate.

## 1. Introduction

**Figure 1:**
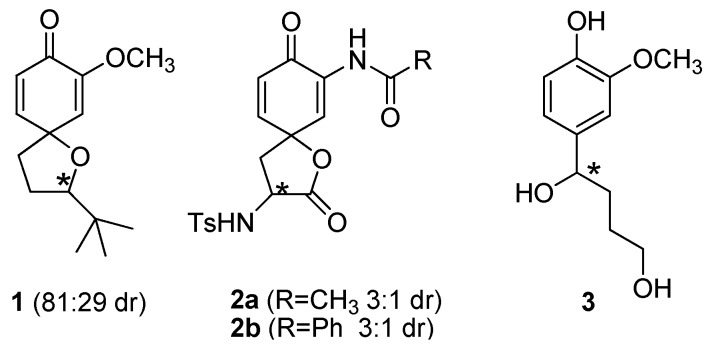
Locations of the chiral centre in spirocompounds and phenol.

In recent years, our work on the oxidative spiroannulation of phenolic derivatives has been focused in part on finding ways to asymmetrically prepare small spirocompounds [1,2,3,4,5]. In 2002 we showed that it was possible to prepare small spiroethers such as **1** (Figure 1) diastereoselectively [1], although the diastereoselectivity (81/19 ratio of diastereomers) was moderate at best. A subsequent investigation resulted in the diastereoselective synthesis of two new spirolactones **2a**,**b** derived from L-3-nitrotyrosine [2], and we were later able to determine the absolute configuration of the spirocarbon in **2** [3]. In the synthesis of spirolactones **2a**,**b**, we observed a slight decrease in the diastereoselectivity (3/1 diastereomeric ratio) of the products formed, which we attributed to the position of the chiral centre on the propionic side chain. As can be seen in Figure 1, the key difference between spirocompounds **1** and **2** is the position of the chiral carbon. In spiroether **1** the chiral carbon is directly linked to the oxygen which acted as the nucleophile in our original investigation [1], while in spirolactones **2** the chiral carbon is found further away from the nucleophilic oxygen. The next logical investigation was to study the effect on the diastereoselectivity that would be associated with a chiral centre located near the future spirocarbon. Hence, we have synthesized the phenolic derivative **3** (Figure 1) to study this effect, as well as study the effect that a longer side chain may have on the diastereoselectivity of this reaction. Our results are reported therein.

## 2. Results and Discussion

**Scheme 1:**
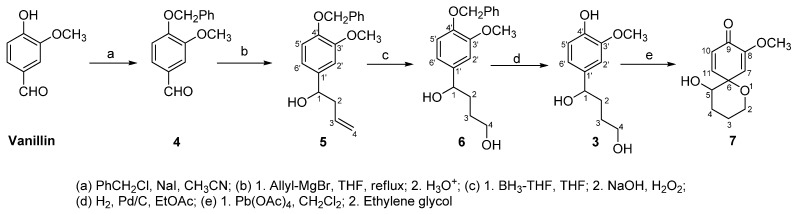
Synthesis of spiroether **7**.

The diastereoselective synthesis of the title compound **7** is shown in Scheme 1. Protection of the phenolic hydroxyl of vanillin was carried out as previously described [1,6] and produced the known benzyloxy vanillin **4** in quantitative yield [7]. This protection was necessary in order to avoid deprotonation with the Grignard reagent in the second step of the synthesis. The subsequent three steps were uneventful, *i.e.*, the formation of the known adduct **5** (91%) [8] was carried out by nucleophilic addition of allylmagnesium bromide to the aldehyde carbonyl in **4**, the anti-Markovnikov hydration of the alkene function in **5** producing **6** (87%), and the deprotection of the phenolic hydroxyl in **6** generating the phenol **3** (93%), all giving the desired product in excellent yields.

**Figure 2:**
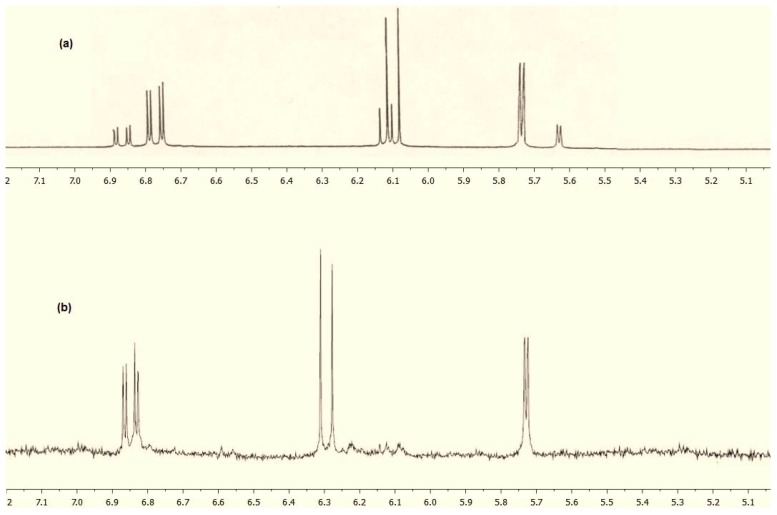
Characteristic portion of the crude ^1^H-NMR spectra of spiroether **1** (**a**) and spiroether **7** (**b**).

However, the synthesis of the spiroether **7** turned out to be more problematic. Our first attempt at transforming **3** to **7** was carried out with phenyliodine bis(trifluoroacetate) (PIFA) in acetone as previously described [2,9,10], but we could not identify the product’s typical signals (two doublets and one doublet-of-doublets between 5.50–7.00 ppm) in the ^1^H-NMR spectrum. These three signals represent the protons of the dienone ring in similar spirocompounds and are usually characteristic of such structure. Figure 2a shows a portion of the ^1^H-NMR spectrum of spiroether **1** as a reference. Other attempts using PIFA (changes in temperature or solvent) ended up giving similar results. We then attempted the spiroannulation with lead tetraacetate in acetone, conditions which gave us good results in previous studies [1,11,12,13,14] and were able to determine that the product had formed based on ^1^H-NMR spectroscopy. However, the amount of material obtained was low (~40%), and the crude ^1^H-NMR spectrum was not clean enough to allow for the determination of the diastereomeric ratio as previously described [1,2,5]. We eventually were able to carry out this transformation and obtained **7** in 61% yield using lead tetraacetate in dichloromethane. However, it should be noted that the product of this reaction appears to decompose easily under the isolation conditions used. For instance, using a long chromatography column packed with silica gel (18 inches in length), we were able to isolate the product in yields ranging from 19–27%. Attempts to increase the yield by adding a small amount of base (Et_3_N) in the eluting solvent (0.1% base was used) increased the yield only marginally to 30–35%. Eventually we found out that vacuum filtration of the product through silica gel gave the best result and spiroether **7** could be obtained in 55–61% yield. In order to confirm the sensitivity of the product to the purification conditions used, 40 mg of purified compound **7** were dissolved in 50% EtOAc/hexanes (10 mL) and 0.5 g of silica gel were added. After stirring at room temperature for 1.5 h, filtering and evaporating the solvent, only 12 mg of the product was recovered (only the signals of spiroether **7** could be observed in the ^1^H-NMR spectrum after this process). We did not attempt to recover the lost material which we assumed was left with the solid support. We have previously observed this type of sensitivity to chromatography with other spirocompounds [9].

**Figure 3:**
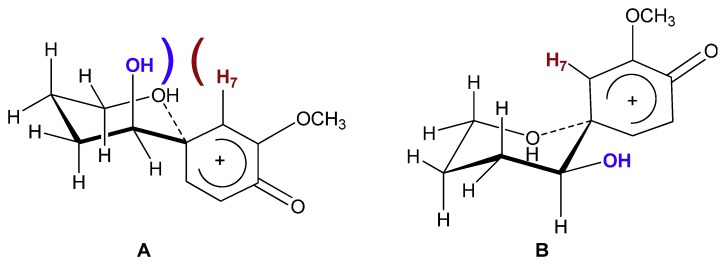
Chair-like conformations of the potential intermediate.

As can be seen from Figure 2, the spiroannulation of **3** produced **7** as a single diastereomer. When compared with the crude ^1^H-NMR spectrum of compound **1** (Figure 2a), all the characteristic signals of the dienone ring of compound **7** (doublet at 5.72, doublet at 6.30 and doublet-of-doublets at 6.85) can be found (Figure 2b), however the second set of signals characteristic of a second diastereomer [as shown in Figure 2a for compound **1**] are absent. While the high diastereoselectivity in this reaction can be attributed in part to the location of the chiral centre in **3**, the fact that a 6-membered ring was formed in structure **7** may also have had an effect on the diastereoselectivity of the reaction. Assuming that: (1) an intermediate phenoxonium ion was produced in the reaction of **3** to **7** as previously reported [4,15] and (2) the approach of the nucleophile took place from a chair-like conformation, one can see that one approach would be more energically preferred. As can be seen in Figure 3, conformation A should not be favored as a strong steric effect can be observed between the axial hydroxyl group on C_1_ of the butanol side chain and H_7_ of the dienone. This steric interaction appears comparable to a 1,3-diaxial interaction on a cyclohexane and the alternative chair-like conformation B should be favored since this steric factor is no longer present given that the hydroxyl group is now in the equatorial position. As a result the conformation of the hydroxyl on C_5_ and the ether oxygen O_1_ would be s*-trans* at the C_5_-C_6_ bond in product **7**.

It should also be noted that while the spiroannulation of phenols leading to spiroethers and spirolactones bearing 5-membered rings is well known and many examples can be found in the literature [16,17], only a few examples of spirocompounds having 6-membered rings as in the structure of **7** [18,19,20] have been reported.

## 3. Experimental

### General

Melting points were determined on a hot stage instrument and are uncorrected. Infrared spectra were recorded either as KBr pellets or neat on a Perkin Elmer System 2000 FTIR. ^1^H-NMR spectra were recorded on a Bruker Fourier 300 spectrometer at 300 MHz and chemical shifts are expressed in ppm using TMS as internal standard. ^13^C-NMR spectra were recorded on a Bruker Fourier 300 spectrometer at 75.4 MHz and chemical shifts are expressed in ppm using the solvent residual signal as internal standard. All spectral assignments were confirmed by COSY, DEPT-135 and HSQC experiments.

*1-(4-Benzyloxy-3-methoxyphenyl)but-3-en-1-ol* (**5**). A solution of allyl magnesium bromide in tetrahydrofuran (2.0 M, 0.05 mL, 0.71 mmol) was added at room temperature under an inert atmosphere (N_2_) to a solution of benzaldehyde **4** (116 mg, 0.47 mmol) in anhydrous tetrahydrofuran (5 mL). The solution was refluxed for 2 h, cooled to room temperature, water (5 mL) was added and the reaction mixture was extracted with ethyl acetate (3 × 10 mL). The organic fractions were combined, washed with brine (10 mL), dried (MgSO_4_) and the solvent was evaporated *in vacuo* to afford a yellow oil (130 mg). Chromatography on silica gel (15% EtOAc/hexanes) afforded a white solid (124 mg, 91%). mp: 58–61 °C; IR (KBr) cm^−1^: 3401; ^1^H-NMR (CDCl_3_) δ: 2.41–2.55 (m, 2H, H_2_), 3.88 (s, 3H, OCH_3_), 4.86–5.06 (m, 3H, H_1_, H_4_), 5.28 (s, 2H, OCH_2_), 5.82–5.95 (m, 1H, H_3_), 6.82 (s, 1H, H_2′_), 6.87–6.95 (m, 2H, H_5′_, H_6′_), 7.31–7.49 (m, 5H, ArH); ^13^C-NMR (CDCl_3_) δ: 40.6 (C_2_), 57.1 (OCH_3_), 71.6 (OCH_2_), 74.9 (C_1_), 113.0 (C_2′_), 114.6 (C_5′_), 117.8 (C_4_), 121.4 (C_6′_), 127.9 (benzyl C_2_, C_3_), 129.9 (benzyl C_4_), 135.1 (C_1′_), 137.2 (C_3_), 138.6 (benzyl C_1_), 148.3 (C_4′_), 152.1 (C_3′_).

*1-(4-Benzyloxy-3-methoxyphenyl)butan-1,4-diol* (**6**). A solution of BH_3_-THF (1 M, 0.7 mL, 0.7 mmol) was added slowly to a cold (0 °C) solution of alkene **5** (132 mg, 0.46 mmol) in anhydrous tetrahydrofuran (5 mL). The solution was stirred at 0 °C for 15 min then at room temperature for 1 h. Aqueous NaOH (1 M, 1 mL, 1 mmol) and 30% hydrogen peroxide (1.9 mL, 6.1 mmol) were added and the resulting solution was stirred at room temperature overnight. The solution was extracted with ethyl acetate (3 × 15 mL), the organic fractions were combined, washed with brine (10 mL), dried (MgSO_4_) and the solvent was evaporated *in vacuo* to afford a tan oil (207 mg). Chromatography on silica gel (30% EtOAc/hexanes) afforded a colorless oil (122 mg, 87%). IR (neat) cm^−1^: 3426, 3039, 1252, 1038; ^1^H-NMR (CDCl_3_) δ: 1.71–1.77 (m, 2H, H_3_), 1.79–1.90 (m, 2H, H_2_), 3.68 (t, 2H, *J* = 6.9 Hz, H_4_), 3.96 (s, 3H, OCH_3_), 4.69 (t, 1H, *J* = 6.8 Hz, H_1_), 5.26 (s, 2H, OCH_2_), 6.91 (s, 1H, H_2′_), 6.91–7.17 (m, 2H, H_5′_, H_6′_), 7.38–7.50 (m, 5H, ArH); ^13^C-NMR (CDCl_3_) δ: 21.4 (C_3_), 34.7 (C_2_), 56.7 (OCH_3_), 64.1 (C_4_), 72.4 (OCH_2_), 73.9 (C_1_), 112.6 (C_2′_), 114.1 (C_5′_), 120.7 (C_6′_), 128.2 (benzyl C_2_, C_3_), 129.1 (benzyl C_4_), 136.2 (C_1′_), 137.7 (benzyl C_1_), 147.5 (C_4′_), 150.1 (C_3′_); Anal. Calc’d for C_18_H_22_O_4_: C 71.50, H 7.33; found C 71.44, H 7.35.

*1-(4-Hydroxy-3-methoxyphenyl)butan-1,4-diol* (**3**). 5% Pd/C (42 mg) was added to a solution of diol **6** (112 mg, 0.37 mmol) in tetrahydrofuran (15 mL). The mixture was placed in a hydrogenator and agitated for 2.5 h under a H_2_ pressure of 30 psi. The mixture was filtered through Celite^®^ using acetone as a washing solvent. The solvent was evaporated *in vacuo* to afford a clear oil (73 mg, 93%). IR (neat) cm^−1^: 3428, 1248,1035; ^1^H-NMR (CDCl_3_) δ: 1.77–185 (m, 2H, H_3_), 1.98–2.08 (m, 2H, H_2_), 3.87–3.95 (overlapping s/m, 5H, OCH_3_, H_4_), 4.11 (t, 1H, *J* = 6.8 Hz, H_1_), 6.79–6.93 (m, 3H, H_2′_, H_5′_, H_6′_); ^13^C-NMR (CDCl_3_) δ: 27.3 (C_3_), 35.6 (C_2_), 57.4 (OCH_3_), 64.7 (C_4_), 75.3 (C_1_), 111.5 (C_2′_), 115.8 (C_5′_), 119.3 (C_6′_), 136.3 (C_1′_), 146.1 (C_4′_), 148.7 (C_3′_): Anal. Calc’d for C_11_H_16_O_4_: C 62.25, H 7.60; found C 62.30, H 7.57.

*5-Hydroxy-8-methoxy-1-oxaspiro{5,5]undeca-7,10-dien-9-one* (**7**). Lead tetraacetate (302 mg, 0.68 mmol) was added at 0 °C to a solution of phenol **3** (68 mg, 0.32 mmol) in dichloromethane (7 mL). The resulting yellow mixture was stirred at 0 °C for 30 min then at room temperature for 4 h. The mixture was filtered through Celite^®^ using dichloromethane as a washing solvent. Ethylene glycol (4 drops) was added to remove the lead residue by chelation and the solution was stirred at room temperature overnight. The mixture was filtered through Celite^®^ and the solvent was evaporated *in vacuo* to give a yellow solid. The product was dissolved in a minimum amount of ethyl acetate and filtered through silica gel using 50% EtOAc/hexanes as eluent to give an off white solid (41 mg, 61%). mp: decomposed; IR (KBr) cm^−1^: 3347, 1646, 1249, 1053; ^1^H-NMR (CDCl_3_) δ: 1.78–1.85 (m, 2H, H_3_), 2.01–2.15 (m, 2H, H_4_), 3.76 (t, 2H, *J* = 7.1 Hz, H_2_), 3.81 (s, 3H, OCH_3_), 4.20–4.26 (m, 1H, H_5_), 5.72 (d, 1H, *J* = 2.9 Hz, H_7_), 6.30 (d, 1H, *J* = 9.1 Hz, H_10_), 6.85 (dd, 1H, *J* = 2.9, 9.1 Hz, H_11_); ^13^C-NMR (CDCl_3_) δ: 25.4 (C_3_), 26.7 (C_4_), 57.8 (OCH_3_), 63.7 (C_2_), 72.4 (C_5_), 81.9 (C_6_), 114.6 (C_7_), 127.1 (C_10_), 149.6 (C_11_), 153.3 (C_8_), 178.1 (C_9_); Anal. Calc’d for C_11_H_14_O_4_: C 62.85, H 6.71; found C 62.81, H 6.73.

## 4. Conclusions

We have successfully carried out the diastereoselective synthesis of a new spiroether in 61% yield and >99% de. While the diastereoselective syntheses of small spirocompounds have been reported before, to our knowledge this represents the first synthesis producing a single diastereomer. We are now investigating the synthesis of optically pure **7** in order to determine the absolute configuration of the new spirocarbon, as well as to confirm our hypothesis for the high level of diastereoselectivity observed in this reaction.
